# Characterizing Biogenic MnOx Produced by *Pseudomonas putida* MnB1 and Its Catalytic Activity towards Water Oxidation

**DOI:** 10.3390/life14020171

**Published:** 2024-01-24

**Authors:** Elisa Morales, Lauren N. Formanski, Sarah E. Shaner, Kari L. Stone

**Affiliations:** 1 Department of Chemistry, Lewis University, Romeoville, IL 60446, USA; elisamorales@lewisu.edu (E.M.); laurennformanski@lewisu.edu (L.N.F.); 2 Department of Chemistry and Physics, Southeast Missouri State University, Cape Girardeau, MO 63701, USA

**Keywords:** Mn(II)-oxidizing bacteria, water oxidation, whole-cell catalysis, multi-copper oxidase, biogenic manganese oxides, BMO, MnB1, acid birnessite

## Abstract

Mn-oxidizing microorganisms oxidize environmental Mn(II), producing Mn(IV) oxides. *Pseudomonas putida* MnB1 is a widely studied organism for the oxidation of manganese(II) to manganese(IV) by a multi-copper oxidase. The biogenic manganese oxides (BMOs) produced by MnB1 and similar organisms have unique properties compared to non-biological manganese oxides. Along with an amorphous, poorly crystalline structure, previous studies have indicated that BMOs have high surface areas and high reactivities. It is also known that abiotic Mn oxides promote oxidation of organics and have been studied for their water oxidation catalytic function. MnB1 was grown and maintained and subsequently transferred to culturing media containing manganese(II) salts to observe the oxidation of manganese(II) to manganese(IV). The structures and compositions of these manganese(IV) oxides were characterized using scanning electron microscopy, energy dispersive X-ray spectroscopy, inductively coupled plasma optical emission spectroscopy, and powder X-ray diffraction, and their properties were assessed regarding catalytic functionality towards water oxidation in comparison to abiotic acid birnessite. Water oxidation was accomplished through the whole-cell catalysis of MnB1, the results for which compare favorably to the water-oxidizing ability of abiotic Mn(IV) oxides.

## 1. Introduction

Manganese oxides (MnOx) are among the strongest oxidants found in the environment [1,2,3]. Manganese (Mn) is a ubiquitous transition-metal element in the surface layer of the Earth and is most often found in its Mn(II), Mn(III), and Mn(IV) oxidation states [3]. The process of converting soluble Mn(II) to insoluble Mn(IV) occurs in many elemental cycles [1,3,4,5]. Although the process of Mn(II)-to-Mn(IV) oxidation is thermodynamically favorable, it is a very slow process without the presence of a catalyst [3]; it has been suggested that the environmental process of Mn oxidation occurs most often in microorganisms such as bacteria and fungi [5]. Some commonly studied organisms for Mn(II) oxidation include *Bacillus* sp. strain SG-14, *Lepothrix discophora* strains SP-1 and SS-64, *Pseudomonas putida* strain GB-1, *Aurantimonas manganoxydans* SI85-9A1 [3,6], *Pseudomonas putida* NRRL B-148787, and *Pseudomonas putida* MnB1 (MnB1) [2,4,7,8,9,10,11,12,13,14,15]. Previous studies have placed the enzymes responsible for biotic Mn(II) oxidation into two broad categories: multi-copper oxidases [1,2,7,8,9,10,16,17,18] and animal heme peroxidases [1,3,11].

*Pseudomonas putida* MnB1 is the manganese-oxidizing organism analyzed in this study. It has been suggested that this organism oxidizes Mn(II) using a multi-copper oxidase enzyme; however, there is no corresponding single-crystal X-ray diffraction structure available [2,7,8,9,10,15,18]. The biogenic manganese oxides (BMOs) formed by MnB1 deposit on the surface of a bacterial cell [8,11]. Although BMOs adopt a layered birnessite-like crystal structure, which is similar to that of abiotic δ-MnO_2_ [4,7,9,10,12,13,14,19,20], they are structurally different from abiotic MnO_2_. The structural differences between BMOs and abiotic MnO_2_ have been investigated using scanning electron microscope (SEM) imaging [2,4,7,13], X-ray diffraction (XRD) spectroscopy [4,10,12,15], energy-dispersive X-ray (EDX) spectroscopy [2,7], and X-ray near-edge absorption spectroscopy (XANES) [9,10,12,13,14,15,16]. Compared to abiotic manganese oxides, BMOs have poor crystallinity [4,7,9,10,12,13,14,15], and owing to their higher surface area, BMOs have been shown to be a more reactive oxidant [4].

Previous studies have investigated the role of MnB1 and its BMOs in the context of soil and water remediation based on the evidence of MnO_2_ acting as a strong oxidizing agent. The high surface areas associated with BMOs suggest they have higher catalytic activity than abiotic MnO_2_; this was the premise of a recent 17α-ethinylestradiol degradation study. The authors of the cited study found that the presence of the manganese-oxidizing bacterium MnB1 degrades 17α-ethinylestradiol 15 times faster than BMO alone [2].

Furthermore, manganese oxides have been of interest for catalytic water oxidation (Equation (1)) and its broader connections to the oxygen evolving complex (OEC) in Photosystem II [17,21,22,23,24,25,26,27,28,29,30,31,32,33,34]. The OEC is the only natural system capable of water oxidation, occurring as part of the photosynthetic process [17,35]. If an OEC-like system was developed on a large scale, it could be a significant source of renewable energy [36]. Based on the idea of manganese oxides being ubiquitous in soil and water, Hocking et al. proposed that a tetranuclear manganese cluster resembling the OEC could be used in water oxidation catalysis [17]. They also suggested that an effective water oxidant would need to have high surface area and high catalytic activity and that the manganese redox cycle resembles biogeochemical manganese cycling [17,37]. A study by Menezes et al. suggested that amorphous MnOx and Mn_2_O_3_ showed a “tremendous enhancement” in photocatalytic oxygen evolution when compared to minerals such as hausmannite (Mn_3_O_4_) and manganosite (MnO) [38]. The amorphous MnOx also had higher catalytic activity than calcium/manganese oxides [38]. The authors suggested that water oxidation activity may have been enhanced by μ-oxo bridges between the Mn centers [38]. Alternatively, Taguchi et al. (2014) theorized that the OEC could be approximated by a Mn complex in the presence of Ca^2+^ ions [39]. The Ca-OH bond on this complex may have also been a facilitator of oxidation by the OEC [39]. Furthermore, the coordination sphere on the OEC-like complex’s “dangling” Mn could be connected to the Ca^2+^ ion through bridging oxo or hydroxo ligands [39].

Water oxidation half reaction: 2 H_2_O → 4 H^+^ + 4 e^−^ + O_2_ (1)

Using nature as an inspiration, biogenic manganese oxides have been prepared by utilizing MnB1 as a source organism. Previous studies have shown different aspects of BMO formation, and yet there is a need to understand the structure and catalytic functionality of biogenic manganese oxides that grant these particles the potential to act as robust oxidation catalysts. In this study, biogenic manganese oxides formed by MnB1 and deposited on the bacterial cell surface were investigated. Specifically, the cell material with BMO on the surface was used without further purification, providing a convenient route to an amorphous material that is an active water oxidation electrocatalyst, presumably due to its high surface area [40,41].

## 2. Materials and Methods

### 2.1. MnB1 Preparation

Cultures of *Pseudomonas putida* (Trevisan) Migula MnB1 supplied by American Type Culture Collection (ATCC #23483) were prepared and grown at room temperature or 30 °C for 3 days. Liquid growth media was prepared according to Jiang et al. [4]. Cultures were added to the liquid growth media and incubated at 24 °C while shaking for four days. Dark-brown or black precipitates formed, indicating the formation of BMOs. Cell material was collected through centrifugation at 3000 rpm for 10 min. Particles were washed with ethanol, and cell material was either freeze-dried or dried in a 60 °C oven overnight.

### 2.2. Material Preparation

Acid birnessite was prepared according to McKenzie using the procedure described by Remucal and coworkers [42,43]. A 10 mL volume of a 0.67 M solution of KMnO_4_ was boiled, and 1.5 mL of 6 M HCl was added dropwise. The solution was boiled for 1 h, cooled to room temperature, and then heated to 60 °C with stirring for ~12 h. The dark-brown solid was collected on a 0.22 μm nylon filter, washed with deionized water, dried under vacuum, and stored in the dark.

### 2.3. Powder X-ray Diffraction

Powder X-ray diffraction (XRD) data of acid birnessite and whole-cell MnB1 samples were collected at room temperature via a Rigaku SmartLab SE diffractometer using a copper anode (with Kα1 = 1.54056 Å and Kα2 = 1.54439 Å) fitted with a nickel Kβ filter. Samples were placed on a zero-background sample holder and analyzed between 2θ 5 and 80° with a step size of 0.01 degrees and scan speed of 1 degree/min.

### 2.4. Scanning Electron Microscopy

Scanning electron microscopy (SEM) with complementary energy-dispersive X-ray spectroscopy data were collected using a JEOL JCM 7000 scanning electron microscope (JEOL Ltd., Tokyo, Japan). Samples for analysis were deposited on atomically flat silica wafers and precoated using 10 nm Au nanoparticles to reduce charging.

### 2.5. Surface Area Analysis

Nitrogen adsorption isotherms were obtained at 77 K using a Quantachrome NOVAtouch LX Surface Area and Pore Size Analyzer (Quantachrome Instruments, Boynton Beach, FL, USA). Samples were prepared by degassing 100 mg acid birnessite for 18 h at 25 °C. Surface area measurements were acquired using the Brunauer–Emmett–Teller (BET) method, and average surface area was obtained.

### 2.6. Determination of Metal Content via ICP-OES

Inductively coupled plasma optical emission spectroscopy was performed with a Perkin Elmer Optima 8000 instrument. To prepare the samples, around 10 mg of acid birnessite or whole-cell MnB1 was dissolved by stirring it in ~5 mL of concentrated HCl for 1 h. The whole-cell MnB1 sample was filtered through a syringe filter, and both solutions were diluted to 10.0 mL in volumetric flasks. These solutions were then subjected to 10-fold and 100-fold dilutions, and all samples (undiluted, 10× diluted, and 100× diluted) were analyzed for Mn, Ca, K, Fe, Cu, and Zn. Calibration standards with concentrations between 1 ppm and 100 ppm were prepared by diluting a 100 ppm multi-element standard (Instrument Calibration Standard 2, SPEX CertiPrep, Metuchen, NJ, USA).

### 2.7. Electrochemical Water Oxidation Studies

Electrochemical water oxidation experiments performed were modified from the procedure described by Stahl et al. [21]. Catalyst inks were prepared by combining 5 mg of catalyst (acid birnessite or whole-cell MnB1), 5 mg of carbon black acetylene (Thermo Scientific Chemicals), 45 mL of 5% wt% Nafion (Thermo Scientific Chemicals), and 350 mL of ethanol. The ink was sonicated for 10 min. Glassy carbon electrodes (3 mm, BASi) were polished with an alumina slurry on a felt pad and rinsed with water and ethanol prior to the deposition of 5 mL of the catalyst ink. The electrode was dried at room temperature for 10 min, followed by 70 °C for 10 min. The electrodes were allowed to cool to room temperature and wetted with a few drops of water before electrochemical analysis.

Electrochemical experiments were performed with a CH Instruments 620E Electrochemical Analyzer using a standard three-electrode cell. An Ag/AgCl reference electrode and platinum wire counter electrode were used in addition to the glassy carbon working electrode with the ink. Linear sweep voltammetry (LSV) was performed in a stirred aqueous 0.1 M NaOH solution at a scan rate of 5 mV/s. Scans were performed by scanning from 0 mV to 1200 mV vs. Ag/AgCl. For each electrode, two scans were performed, and in all cases, data from the second scan were used. Reported LSV data are the average of three electrodes.

## 3. Results

MnOx is a strong oxidant and has been shown to have applications in catalysis, environmental remediation, and water oxidation [42,44,45]. For example, Guot et al. recently reported the growth of manganese oxides on an electrode in order to construct a fuel cell [46]. Using nature as an inspiration for catalysis, we utilized biogenic manganese oxides from *Pseudomonas putida* strain MnB1 as a Mn(II)-oxidizing organism.

The putative Mn(II)-oxidizing enzyme in MnB1 resembles a multi-copper oxidase due to its sequence homology with respect to other well-known multi-copper oxidases such as laccase. The structure of the MnB1 Mn(II)-oxidizing enzyme has not been experimentally determined, but its sequence has been identified, CumA, which shows a high degree of sequence homology to other multi-copper oxidases. CumA is not a membrane-bound protein, and it is localized internally. The BMO material accumulates on the cell surface and can then be secreted into the environment. 

**Figure 1:**
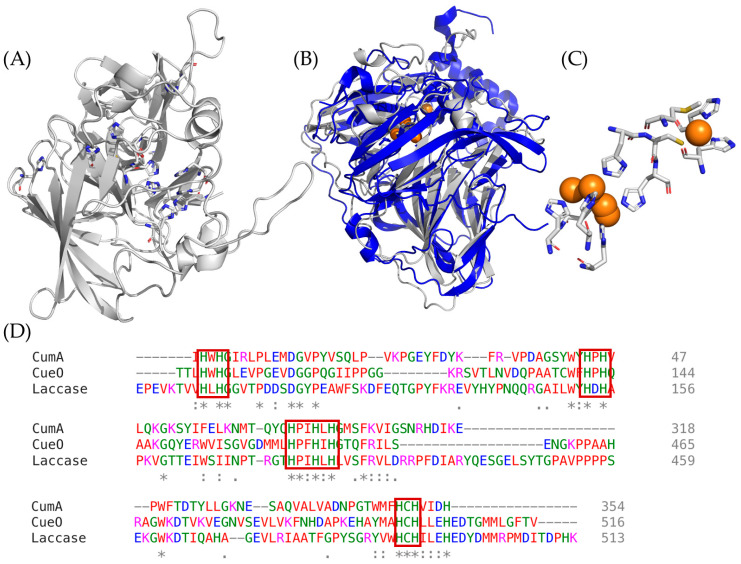
Comparison of the theoretical structure of CumA from *Pseudomonas putida* and other well-studied multi-copper oxidases [18]. (**A**) Proposed structure of CumA determined in AlphaFold [47,48]; (**B**) overlay of the calculated structure of CumA and Laccase from Bacillus subtilis; (**C**) active site of Laccase; (**D**) protein sequence alignments of CumA (unknown structure). CueO a Mn(II)-oxidizing multi-copper oxidase from *Escherichia coli* strain K12 and Laccase. (*) indicates conserved amino acids, (:) indicates semi-conserved residues, (.) indicates an evolutionary divergence of residues. Letter colors indicate the type of amino acid: green (polar), red (nonpolar), blue (acidic), and pink (basic). Red boxes highlight the active sites that bind copper ions and are conserved in all multi-copper oxidases.

Utilizing AlphaFold as a structural guide, a structure can be predicted with a high degree of certainty [47,48]. AlphaFold, developed by DeepMind and EMBL-EBI, is an artificial intelligence algorithm that is continually adding proteins to a protein structure database. This technology is based on an artificial intelligence system developed through a partnership between DeepMind and EMBL’s European Bioinformatics Institute. The database currently contains 3D structures of 200 million proteins, integrating itself into Uniprot, the standard repository of protein sequences and annotations. AlphaFold is a validated neural-network-based model that demonstrates high accuracy in identifying a structural prediction that correlates well to experimental structures and outperforms other methods of structural prediction. Figure 1 depicts a sequence and structural comparison between the Mn(II)-oxidizing enzyme from MnB1 (CumA) and other known multi-copper oxidases [47,49,50,51]. The theoretical structure of the MnB1-derived enzyme is shown in Figure 1, where it is overlaid with laccase, depicting their structural similarities. Figure 1D shows the sequence alignment of the Mn(II)-oxidizing enzyme from MnB1, CueO, a known multi-copper oxidase from *Escherichia coli* strain K12, and laccase from *Bacillus subtilis*, where the active site residues are highlighted, indicating that the active site structure and therefore reactivities are similar. All highly conserved residues are denoted with an asterisk. HWHG, WHPH, HPIHLH, and HCH are among the sequence regions that appear in all the examples of multi-copper oxidases, and these sequences are both catalytically and structurally relevant to multi-copper oxidases. Based on this analysis, the Mn(II)-oxidizing enzyme from MnB1 was presumed to be a multi-copper oxidase.

To prepare the biogenic manganese oxide samples, *Pseudomonas putida* MnB1 cultures were incubated with liquid growth media, and dark-brown or black BMO precipitates formed. After 4 days, the cultures showed the maximum amount of BMO formation. Cell material was collected, washed, and freeze-dried, and this material (whole-cell BMO) was used without further workup or purification. Whole-cell BMO from MnB1 was analyzed via powder X-ray diffraction (XRD), scanning electron microscopy (SEM), and energy dispersive X-ray spectroscopy (EDX) mapping, along with inductively coupled plasma optical emission spectroscopy (ICP-OES). Therefore, the size, morphology, and composition of the BMOs in the whole-cell material were thoroughly investigated in this study. The BMO material was compared to the abiotic MnO_2_ acid birnessite, which is a layered manganese oxide with the formula MnO_2_ that has been theorized to have a similar structure to BMO. There are several terms used, often interchangeably, to name synthetic layered MnOx materials, including δ-MnO_2_, birnessite, and acid birnessite, and they are often differentiated by the synthetic route used to prepare them [52]. The material used in this study is usually referred to as acid birnessite, which was prepared from KMnO_4_ via the method described by McKenzie [42,43].

The elemental composition of the whole-cell BMO and acid birnessite samples are interesting with respect to determining the amount of Mn as well as the presence of other metal ions. ICP-OES analyses were performed to determine their metal compositions, and the samples were tested for seven metals: Mn, Ca, K, Mg, Fe, Cu, and Zn. The results of the ICP-OES analyses are shown in Table 1. Whole-cell BMO was shown to contain 7.0% manganese and 0.6% calcium, with no significant amounts of other metals. The remainder of the mass is presumed to be primarily organic cell material. The acid birnessite sample is 44.6% manganese and 5.0% potassium by mass. The incorporation of a significant amount of potassium is consistent with the synthesis of this material from a potassium permanganate solution. These results were compared with images generated by EDX mapping.

**Figure 2:**
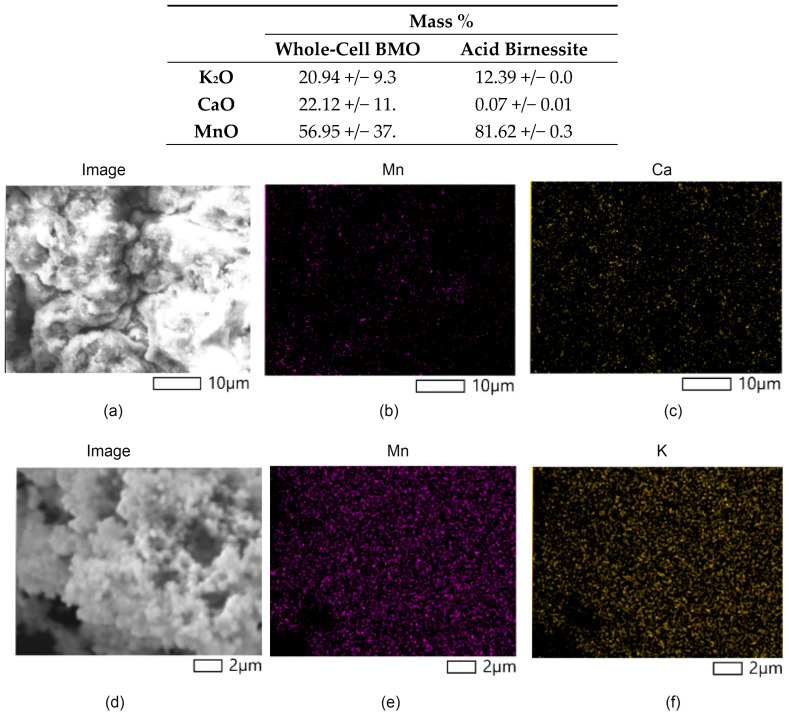
SEM and EDX analysis of whole-cell BMO (**top**) and acid birnessite (**bottom**). (**a**) SEM image of whole-cell BMO, (**b**) Mn content of whole-cell BMO, (**c**) Ca content of whole-cell BMO, (**d**) SEM image of acid birnessite, (**e**) Mn content of acid birnessite, and (**f**) K content of acid birnessite. Scale bars are shown in the figure.

SEM is a surface technique that allows for the visualization of the structures of particles and, with the addition of the complementary technique EDX, the mapping of elements on the surface. An array of elements was analyzed, but only Mg, Ca, Mn, Al, and K showed significant contributions. Other than manganese, potassium is most abundant in the acid birnessite, and calcium is most abundant in the BMO material. SEM images of whole-cell BMO and acid birnessite are shown in Figure 2. The top image represents the values obtained from the EDX measurements of the surfaces of whole-cell BMO from MnB1 and acid birnessite. The SEM images reveal amorphous materials for both acid birnessite and whole-cell BMO. These materials were also analyzed using XRD, showing that there is indeed a slight degree of crystallinity in these materials (vide infra).

Whole-cell BMO images are displayed in Figure 2a–c, and acid birnessite images are displayed in Figure 2d–f. Elemental mapping of manganese is shown for both samples, and mapping of calcium in whole-cell BMO from MnB1 and potassium in acid birnessite is also presented. The scale bars indicate the relative sizes of the amorphous materials. As also shown from the ICP-OES measurements, the EDX maps reveal that calcium was the highest-concentrated 2+ ion in whole-cell BMO, with Mg^2+^ being the second highest and four times less concentrated than calcium. The EDX values differ from the ICP measurements of the whole-cell BMO because they were analyzed as oxides on the surface of the material while the ICP measurements represent the mass percent of the whole sample. In addition, the whole-cell BMO image contains a substantial amount of organic material since the image was produced without the removal of organics. These images reveal the relative size and amorphous structure of both the biogenic and abiotic MnO_2_ materials. 

**Figure 3:**
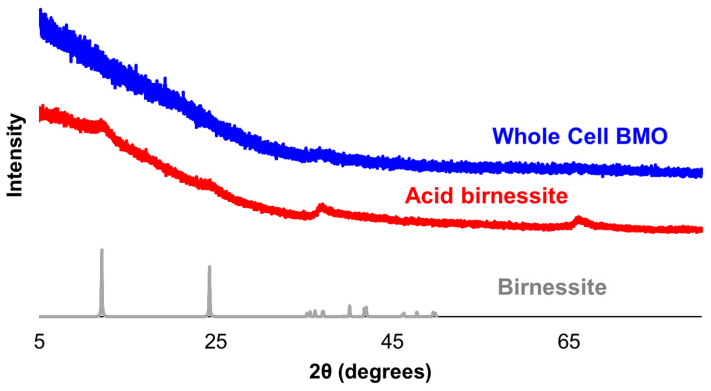
Powder X-ray diffraction patterns of whole-cell BMO from MnB1 and acid birnessite samples. The XRD pattern of a layered Mn oxide, birnessite (Crystallography Open Database ID entry: 9013650), is provided for reference.

The structures of the whole-cell BMO from the MnB1 sample and acid birnessite were investigated using powder X-ray diffraction, and the diffraction patterns are shown in Figure 3. As indicated by the lack of defined diffraction peaks, the whole-cell BMO material is quite amorphous. The acid birnessite sample is also fairly amorphous, but peaks that are consistent with layered Mn oxide structures are observable. The surface area of the acid birnessite sample was measured using the Brunauer–Emmett–Teller method and determined to be 20.3 m^2^/g. While the surface area of the whole-cell BMO material was not measured via BET because the presence of cell material would limit its usefulness in assessing the BMO, the SEM and XRD data indicate that the BMO produced by MnB1 likely has a large surface area.

**Figure 4:**
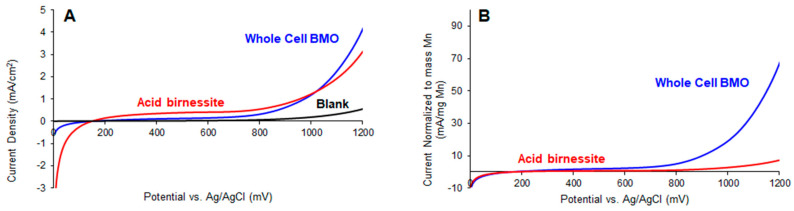
Linear sweep voltammetry at a scan rate of 5 mV/s in 0.1 M NaOH solution of whole-cell BMO and acid birnessite catalyst inks. (**A**) Current density normalized to the geometric surface area of the glassy carbon working electrode. (**B**) Current normalized to the mass of Mn on the electrode as determined via ICP-OES.

The suitability of using whole-cell BMO as an electrocatalyst for the water oxidation half reaction was studied using linear sweep voltammetry (LSV), and the activity of this material was compared to that of the abiotic acid birnessite. LSV experiments were conducted under basic conditions in 0.1 M NaOH solutions at a scan rate of 5 mV/s. The LSV results for acid birnessite in this study are very similar to those reported for other layered MnO_2_ materials, such as δ-MnO_2_ [21]. Under these conditions, the current densities reached by the whole-cell BMO were similar, and slightly higher, than those of acid birnessite (Figure 4A). This result is particularly interesting because while the same mass of sample was tested for each material, the whole-cell BMO contained much less Mn (7%) than the acid birnessite sample (45%). To highlight this result, the currents in the linear sweep voltammograms were normalized based on the amount of Mn in each sample as determined via ICP-OES measurements. Figure 4B shows the LSV data, with currents reported in milliamps per milligram of Mn, and the current normalized to mass of Mn is nearly 10 times higher for whole-cell BMO than that of acid birnessite at 1200 mV vs. Ag/AgCl (68 mA/mg vs. 7 mA/mg). This indicates that the whole-cell BMO is a more potent catalyst.

## 4. Conclusions

In this study, biogenic manganese oxides were produced by the Mn(II)-oxidizing organism *Pseudomonas putida* MnB1, and the whole-cell material comprising the BMO and the cell material were characterized using XRD, SEM-EDX, and ICP-OES. These analyses indicated the formation of an amorphous MnOx material that included a significant amount of calcium. The BMO cell material also showed activity towards water oxidation. These data were compared to the abiotic manganese oxide acid birnessite due to studies showing that these manganese oxides might have similar structural characteristics.

The appearance of calcium as a metal that has a substantial composition in the MnOx particles inspired our interest in comparison to the oxygen-evolving complex in photosynthesizing organisms. The Mn/Ca ratio in whole-cell BMO tended towards ~90:10 in all of our analyses (ICP-OES and EDX mapping). Of note, there was a larger concentration of calcium ions than that of metals other than manganese. While the intercalation of Ca^2+^ into the pores of manganese oxide particles is a known phenomenon, the role of Ca^2+^ in water oxidation constitutes a question that remains to be addressed. For comparison, the metal composition of the OEC is a ~85/15 ratio of Mn/Ca. Previous studies have shown that a calcium ion is essential for water oxidation in the OEC and, later, that calcium is a structural component of the cluster [35]. A broadly accepted mechanism of O-O bond formation involves the reaction of an electrophilic Mn-oxo and a metal-bound nucleophile [2]. An extension of this theory involves naming the nucleophile as the Ca-OH unit because it is redox-inactive and can act as a nucleophile, which is essential in the formation of an O-O bond, where manganese ions alone would facilitate one-electron, or non-productive, processes, and these must be avoided to make the O-O bond [39]. It is also known that metals can intercalate into BMOs, and some researchers have exploited this ability by using them to remediate heavy metals from natural waters [20]. We hypothesize that the presence of calcium that can easily intercalate into holes in particles may serve as a contributing component for water oxidation. Investigations into increasing the intercalation of calcium into BMO materials by varying the growth conditions of MnB1 are ongoing in our laboratory.

The catalytic activity of the whole-cell BMO from MnB1 material for water oxidation was tested electrochemically using linear sweep voltammetry. This material showed activity similar to that of the related MnOx acid birnessite. However, because much of the whole-cell BMO material is organic matter, this comparable activity is derived from a much smaller amount of Mn. When the current is normalized to the amount of Mn in each sample, the whole-cell MnB1 material looks quite promising as a water oxidation catalyst. The high per-Mn activity observed is likely a result of the high surface area of the material, which allows more Mn sites to be electrochemically accessible. Although the surface area of the whole-cell BMO material was not measurable via BET, the XRD and SEM results support the conclusion that an amorphous material was prepared. The formation of an amorphous, high-surface-area material might be facilitated by the presence of the cells on which the BMO is deposited. Previous studies have shown that layered, amorphous MnOx materials tend to be the most active water oxidation electrocatalysts [30]. In the future, studies will be performed to measure the Faradaic efficiency of O_2_ production by whole-cell BMO material. In addition to their application as water oxidation catalysts, BMOs, even in samples with an intact organism, would likely also show promise in the remediation of organic pollutants and prove to be a sustainable tool for bioremediation. Future studies will include attempting to grow MnB1 directly on electrodes and utilizing whole-cell BMO for other oxidation processes.

## Figures and Tables

**Table 1:** Composition of whole-cell BMO from MnB1 and acid birnessite via ICP-OES. Samples were dissolved in concentrated HCl and tested for manganese, calcium, potassium, magnesium, iron, copper, and zinc.

	Mass Percent of Element in Sample						
	Mn	Ca	K	Mg	Fe	Cu	Zn
Whole-cell BMO	7.0%	0.6%	0.3%	0.3%	0.1%	0.2%	0%
Acid birnessite	44.6%	0%	5.0%	0%	0%	0.1%	0%

## Data Availability

The raw data supporting the conclusions of this article will be made available by the authors on request.
