# Correction: Morales et al. Characterizing Biogenic MnOx Produced by *Pseudomonas putida* MnB1 and Its Catalytic Activity towards Water Oxidation. *Life* 2024, *14*, 171

**DOI:** 10.3390/life14060767

**Published:** 2024-06-17

**Authors:** Elisa Morales, Lauren N. Formanski, Sarah E. Shaner, Kari L. Stone

**Affiliations:** 1 Department of Chemistry, Lewis University, Romeoville, IL 60446, USA; elisamorales@lewisu.edu (E.M.); laurennformanski@lewisu.edu (L.N.F.); 2 Department of Chemistry and Physics, Southeast Missouri State University, Cape Girardeau, MO 63701, USA

## Addition of an Author

“Lauren N. Formanski” was not included as an author in the original publication [1]. The corrected Author Contributions statement appears here. The authors state that the scientific conclusions are unaffected. This correction was approved by the Academic Editor. The original publication has also been updated.

**“Insert new Author Contributions Statement”**
